# Editorial: The Intersections of Economic Insecurity, Non-Standard Employment and Gender in Southern Europe

**DOI:** 10.3389/fsoc.2021.730671

**Published:** 2021-07-14

**Authors:** Lara Maestripieri, Christiana Ierodiakonou

**Affiliations:** ^1^ Politecnico di Milano, Milan, Italy; ^2^ University of Cyprus, Nicosia, Cyprus

**Keywords:** economic insecurity, non-standard work, southern europe, intersectionality, women’s work

## Background of the Special Issue

This collection of papers examines economic insecurity in relation to non-standard employment from a gender perspective and with an emphasis on the southern European context. Given changes in the broader socio-economic and political contexts, as well as changes in the modes and patterns of working, this special issue provides insights into the complex ways that gender relates to these changes within southern European countries. Albeit small, this collection of articles adds to extant literature and helps move relevant research forward in two main ways. First, it highlights the need to enrich relevant research with the incorporation of context, and particularly of under-researched and idiosyncratic contexts such as the southern European one. Second, it denotes the importance of adopting interdisciplinary approaches and methods of research if we are to better understand how gender and other dimensions of identity –and exclusion –interact and affect working modes, experiences and outcomes.

Economic insecurity is an issue that has received increasing academic attention in general, but especially over the last years, following the 2008 global economic crisis. Economic insecurity can be defined as ‘the anxiety produced by the possible exposure to adverse economic events and by the anticipation of the difficulty to recover from them’ (Bossert and D’Ambrosio, 2013, p. 1018). It is therefore a condition experienced at the micro-level, subjectively perceived by individuals who feel they are in a vulnerable and insecure position, but is nonetheless triggered by rather ‘objective’ conditions, such as financial strain and difficulties in covering basic needs experienced by households. Economic insecurity therefore relates to risk(s) of livelihood such as economic loss, unemployment or under-employment (Kopasker et al., 2018). Indeed, economic insecurity is often associated with labor market insecurity and therefore also with issues such as unemployment, tenure, working hours and compensation (Misra, 2021; Western et al., 2012). Nonetheless, many other adverse events are associated with economic insecurity, including events relating to family, health (Western et al., 2012) and classes (Ranci et al., 2017).

Vulnerability to economic insecurity seems to be closely tied to changes in employment relations and working conditions. As Kopasker et al. (2018) rightly argue, such a noticeable change is that non-standard employment through precarious contract arrangements is becoming increasingly normalized. For Hollister (2011), this represents a new employment narrative that gradually transforms employment relations to short-term, contractual agreements. How does this then relate to experiences of economic insecurity and what socio-economic consequences are individuals faced with if we consider evidence associating insecurity with multiple negative effects on mental and physical health?

There seems to be a common agreement that, as labor markets are changing, they are becoming more precarious (Gutiérrez-Barbarrusa, 2016; Misra, 2021). As per Lorey (2015), precariousness relates broadly to insecurity, vulnerability, uncertainty and endangerment. However, these negative effects are disproportionately distributed and affect specific groups of people more than others. This is primarily due to pre-existing structures and systems of socio-economic inequalities (Maroto et al., 2019). Such structures are historically and culturally embedded and therefore become hard to shift boundaries through which inequalities are typically reproduced, making specific groups structurally vulnerable and thus more likely to run elevated risks in labor markets and in life (Whittle et al., 2020). For instance, relevant studies show a link between women’s labor market condition and exposure to vulnerability determined by economic insecurity. At the same time, though, women’s economic insecurity is not solely affected by labor market participation, but from a combination of multiple interconnecting factors (Kasearu et al., 2017). To indicate, Ierodiakonou (2014) explored the work-family trajectories of three birth cohorts of women in Cyprus to conclude that women’s agency in making work-related decisions remained, over time, limited by several broader structures and that traditional social norms operated through class, gender and family relationships in complex ways.

Women, as well as members of other minority groups, experience higher levels of economic insecurity since they are more likely to find themselves in a disadvantaged position in the labor market, in relation to their counterparts (Kopasker et al., 2018; Maroto et al., 2019). Up to now, however, the debate on economic insecurity has largely taken the household as the minimal unit to investigate these dynamics: as such, gender dynamics over the phenomenon have been covered by couple relationships (e.g., Mauno and Kinnunen, 2002; Western et al., 2012). An individual-based investigation of economic insecurity allows investigating to what extent gender influence the subjective perception of economic insecurity in its relationship with labor precariousness, going beyond the current debate.

Relatedly, we know little about what happens when gender interacts with other socially constructed statuses and how those interactions shape different experiences of disadvantage, employment experiences and paths to non-standard, precarious work and economic insecurity (Lavaysse et al., 2018). Socially constructed statuses interact and their intersectionality creates mechanisms of accumulated disadvantage and inequality, making some individuals and groups more vulnerable to economic insecurity compared to others (Maroto et al., 2019; Misra, 2021). So while women in general may be more likely to have non-standard employment compared to men, where gender interacts with other dimensions–such as race, migration status, social class or disability–different hierarchies of disadvantage are created (Maroto et al., 2019). This reveals thus the complexity involved in understanding these issues and the need to move from a gender perspective to an intersectional approach in order to understand how insecurity and disadvantage are multiplied and reproduced across different axes. To quote (Misra (2021), pp. 2–3), ‘gender, nationality, and race are implicated in nonstandard employment relations, job insecurity, economic insecurity, and subjective well-being, as well as policy solutions to all of these’.

For this special issue, another one of those axes is geography: the need to generate more research insights from under-researched contexts such as the one of southern European countries. Southern Europe deviates substantially from the Anglo-Saxon context where most research evidence comes from Ferrera (1996). Furthermore, Southern Europe was most severely affected by the economic crisis, which has had profound effects on labor markets and other institutions, putting a large proportion of the population in a state of long-term economic insecurity. Though in different and varying degrees, southern European countries have experienced increases in non-standard, casual and informal work that have changed the work and employment patterns of all individuals, more so of women. Coupled with changes in socio-economic policies, women’s large and often involuntary share in precarious forms of work and employment places women at a state of higher economic insecurity.

Through comparative research, Kasearu et al. (2017), for example, conclude that there are different configurations of factors that explain women’s exposure to economic insecurity, clearly indicating that, even within Europe, specific contextual circumstances affect individual vulnerability. Maestripieri and León (2019) have demonstrated the increasing exposure of women to involuntary non-standard employment in Southern Europe, which is indeed one of the main determinants of the economic insecurity as previously argued. The 2008 financial crisis has worsened this situation and we might expect a corresponding worsening over women’s condition as a consequence of the Covid19 disruption (see Maestripieri in this issue). On a related note, Gutiérrez-Barbarrusa (2016) concludes that, even though precarious employment generally increased following the 2008 financial crisis, the drivers of this increase varied among countries: while poverty drove to increases of precarious work in deregulated markets, insecurity was the main driver in southern European countries.

Future research–especially those exploring the socio and economic consequences of the Covid19 pandemics–should go beyond the household approach to economic insecurity and focusing on the individual condition of insecurity. An intersectional analytical framework allows scholars to disentangle the concomitant effect of the different axes of inequalities, such as gender, migrant background and locality–as done by the contributions in this special issue.

## Contributions in the Special Issue

Articles in this collection are diverse and come from different perspectives and contexts, but all contend with the aforementioned key concepts, albeit to different extents: economic insecurity, non-standard employment, gender and intersectionality.

Bracci and Riva offer a cross-country comparative analysis using data from the European Working Conditions Survey. The authors approach job insecurity as the risk of involuntary job loss and investigate its links to anxiety from a gender perspective. In so doing, they find that women are overall more vulnerable to experience anxiety compared to men when perceiving their jobs as insecure. Interestingly, though, their analyses did not reveal ‘gender’ as a statistically significant moderator in the relationship between job insecurity and anxiety. What’s more, despite some country differences that Bracci and Riva discuss, the association between job insecurity and anxiety appears to be fairly stable across most European countries. This could suggest that the macro-level national context does not have the impact one might initially assume. Instead, it could be that individual feelings of insecurity unequivocally relate to anxiety, regardless of the broader context. In this sense, instead of focusing on institutional policies or welfare protection for employees, we should place emphasis on improving employment conditions and job security in order to reduce feelings of anxiety and other mental health implications. These findings can guide relevant research as it moves forward. Researchers may try to untangle the complexity surrounding these issues by including more and/or different macro-level dimensions. Examples could include relevant legislation, cultural values and norms, equality indices, welfare state investments, etc. Further studies could also include dimensions of identity in addition to gender, to offer an even more complete picture around associations between job insecurity and anxiety, following a more interdisciplinary approach.

Such an approach is more evident in the contribution of Gewinner and Salvino, who use a pluralistic methodology that combines interview and online data with observation and judicial material to explore how women from the former Soviet Union experience economic insecurity as migrants in three different countries: Germany, Italy and Spain. Employing an intersectional approach, the analysis offered considers aspects such as age, social background and education to understand how these migrant women cope with feelings of economic insecurity. Through a ground research approach, this article provides critical insights into the different meanings that economic insecurity may have for different individuals. Here, analysis and interpretation are carefully and fully contextualized and the authors put forth the idea that perceptions of insecurity relate to deeper and broader concepts and values, thus relating to institutional and cultural dimensions.

Finally, Maestripieri focuses on intersectionality specifically in relation to the ongoing Covid 19 pandemic in an article that highlights how a crisis such as this places specific groups at an elevated risk –in terms of their health and economic insecurity –bringing forward issues related to structural vulnerability. Maestripieri argues that the pandemic is an intersectional phenomenon relating to multiple interconnecting structures of inequality and therefore understanding its determinants and consequences necessitates an intersectional framework.

Taken together, contributions in this special issue may be different, but come together in a collection that generally calls for deeper, more elaborated and more complex explorations of a social phenomenon such as economic insecurity that is triggering new forms of inequalities in contemporary societies.
